# Longitudinal study of habits leading to malocclusion development in childhood

**DOI:** 10.1186/1472-6831-14-96

**Published:** 2014-08-04

**Authors:** Suzely Adas Saliba Moimaz, Artênio José Ísper Garbin, Arinilson Moreira Chaves Lima, Luiz Fernando Lolli, Orlando Saliba, Cléa Adas Saliba Garbin

**Affiliations:** 1 Department of Infant and Social Dentistry, Araçatuba School of Dentistry - UNESP, José Bonifácio Street, 1193. Vila Mendonça, Zip Code 16015-050 Araçatuba-São Paulo, Brazil; 2 Graduate Program in Preventive and Social Dentistry, Araçatuba School of Dentistry - UNESP, Araçatuba-São Paulo, Brazil

**Keywords:** Oral health, Malocclusion, Infant, Longitudinal studies

## Abstract

**Background:**

The increased prevalence of malocclusions represents a secular trend attributed to the interaction of genetic and environmental factors. The analysis of factors related to the causes of these changes is essential for planning public health policies aimed at preventing and clinically intercepting malocclusion. This study investigated the sucking habits, nocturnal mouth breathing, as well as the relation of these factors with malocclusion.

**Methods:**

This is a longitudinal study in which 80 mother-child pairs were monitored from the beginning of pregnancy to the 30^th^ month after childbirth. Home visits for interviews with the mothers were made on the 12^th^, 18^th^ and 30^th^ months of age. Finger sucking, pacifier sucking, bottle feeding, breastfeeding and nocturnal mouth breathing, were the variables studies. On the 30^th^ month, clinical examinations were performed for overjet, overbite and posterior crossbite. A previously calibrated single examiner (Kappa coefficient = 0.92) was responsible for all examinations. Data were analyzed using the chi-squared or Fisher’s exact tests, at a significance level of 5%.

**Results:**

Bottle feeding was the most prevalent habit at 12, 18 and 30 months (87.5%; 90% and 96.25%, respectively). Breastfeeding was 40%, 25% and 12.50% at 12, 18 and 30 months, respectively. Nearly 70% of the children in this study had some sort of malocclusion. Pacifier sucking habit at 12, 18 and 30 months of age was associated with overjet and open bite; and at 30 months, an association with overbite was also observed. Finger sucking habit and breastfeeding at 12, 18 and 30 months were also associated with overjet and open bite. The posterior crossbite was associated with bottle feeding at 12 and 30 months, and nocturnal mouth breathers at 12 and 18 months.

**Conclusions:**

Sucking habits, low rates of breastfeeding, and nocturnal mouth breathing were risk factors for malocclusion.

## Background

The increased prevalence of malocclusions represents a secular trend attributed to the interaction of genetic and environmental factors [1]. Malocclusion is a growth and development deviation, mainly of the muscles and jaw bones during childhood and adolescence, and may be related to harmful habits of early childhood [2]. The etiology of malocclusion has genetic and environmental components [3], and its study is essential for the success of orthodontic treatment since a prerequisite for correction is the elimination of the causes [4]. There are controversies regarding the causes of malocclusion in primary dentition and whether or not these are predictive of malocclusion in the permanent dentition [4,5]. The analysis of factors related to the causes of malocclusion is very important for planning public health policies aimed at preventing and clinically intercepting this health problem [1]. In view of the increasing interest in the early diagnosis and treatment of malocclusions and the corresponding emphasis on the preventive procedures, further information is needed [4].

“Habit” is a practice acquired by the frequent repetition of the same act, which occurs consciously at first, then unconsciously [6]. Pacifier sucking, followed by finger sucking are the most common harmful habits in childhood, mainly from 0 to 3 years, due to the process of development and world discovery [7]. However, such oral habits are major risk factors for malocclusion in deciduous teeth, and their harmful consequences have been reported in several epidemiological studies [5,8-10]. Another condition often pointed out in studies as a risk factor for the onset of malocclusion and facial problems is mouth breathing, a habit that occurs most commonly during sleep [11,12].

Breastfeeding is seen as a determining factor for proper craniofacial development, because it promotes intense exercise of the orofacial muscles, favorably stimulating the functions of breathing, swallowing, mastication and phonation [13]. However, replacing breastfeeding with bottle feeding is mentioned in the literature as a factor with consequences for children's health [14].

According to Fernandes et al. [15], the first orthodontic consultation should take place as soon as the child completes their deciduous arch. Children at the 30^th^ and 36^th^ months of age can provide evidence of occlusal imbalance, which facilitates prior preventive planning in order to avoid the advance of an adverse occlusal condition [15]. In a study with Brazilian children aged 1-5 years, 76.32% had never visited a dentist [16].

In developing countries, there is limited access to dental services and patients often seek only emergency treatment [4]. Given the restraints due to high costs of orthodontic services and lack of publicly funded dental treatment programs in these countries, it becomes increasingly important to recognize the need for orthodontic treatment according to the severity and to identify modifiable factors that can be addressed through preventive and interceptive orthodontics [17]. After finding a strong association between sucking habits and malocclusion in deciduous dentition, Katz et al. [9] emphasized the need for longitudinal studies to better support the clinical decisions. Few longitudinal studies associating malocclusion and sucking habits in children until 30 months of age were found in the literature. The access to children in early childhood can be difficult due to the fact that many of them are too young to attend day care centers or schools [4]. This study sought to overcome this difficulty, presenting as a differential the follow-up home visit and a longitudinal approach. Thus, sucking habits, nocturnal mouth breathing and malocclusion were investigated, as well as the association of these factors, in order to provide information to help fill the gap about the topic in early childhood.

## Methods

### Ethics

This research formed part of “The Impact of Care in the Practice of Maternal Breastfeeding and Oral Health on the Mother-Child Binome,” developed by the Graduate Program in Preventive and Social Dentistry of São Paulo State University (UNESP). First, a research project was submitted and approved by the Ethics Committee on Research Involving Humans of the Araçatuba School of Dentistry - UNESP.

### Sample

This was a prospective cohort study of the development of malocclusion in childhood. The sample consisted of all pregnant women (and their children after birth) in a municipality of the state of São Paulo, Brazil, who were enrolled in a public program for the monitoring of prenatal care, at baseline, totalling 120 pairs. Women who provided informed consent were included in the study. Of the total, 40 women chose not to participate, or have moved address, which is common for this type of study, so that the final sample considered was 80 mother-child pairs. For this finite population (120), considering 70% of malocclusion frequency in literature [18,19], confidence limits 5%, and design effect 1; for a confidence level of 80%, commonly used, the sample size should be 65. In this study, with final sample of 80 participants, the confidence level was greater than 90%.

### Data collection

For this research, data collection occurred on the 12^th^, 18^th^ and 30^th^ months after the children’s birth, at the houses of participating mothers, at previously scheduled times and dates, which did not interfere with the families’ well-being. Mothers received the necessary clarifications about how the study would be conducted, but care was taken not to influence them to give socially desirable responses in the phase of the data collection. Interviews with the mothers were conducted using a semi-structured questionnaire. A pilot study was carried out with 20 female participants in a project about dental care during pregnancy in the Araçatuba School of Dentistry - UNESP, in order to validate the instrument and calibrate the researcher. During pregnancy, in the first part of the major research, mothers received orientation about oral health and deleterious habits. In the visits, mothers were questioned whether they were breastfeeding, the incidence of other sucking habits (pacifier, finger, bottle) and the child’s nocturnal breathing mode (nose or mouth). Pacifier and finger sucking was considered a habit when the child did it for more than two hours a day. Thus, avoided in the results were cases in which this type of suction was seldom practiced, as detected in the pilot study. Breastfeeding was considered present when the child was fed at least once a day, even when taking other sources of nutrients. The same criterion was applied to the bottle habit. Children who slept with their mouth open were considered nocturnal mouth breathers.

Data collection for this study occurred between November 2008 and May 2010. Intra-oral examinations were performed on children at the 30^th^ month of age to detect signs of malocclusion. A single examiner (doctoral student) previously calibrated (Kappa coefficient = 0.92) and duly parameterized (jackets, caps, gloves and mask), was responsible for all examinations, with a World Health Organization (WHO) periodontal probe, dental mirror and gauzes, under ambient light. All children examined had 20 deciduous teeth in the mouth.

The outcomes were:

Overjet: Obtained by measuring the horizontal ratio between the upper and lower incisors with the teeth in occlusion. The distance between the incisal edge of most prominent upper incisor and the buccal surface of the corresponding lower incisor was measured with the periodontal probe parallel to the occlusal plane. This distance was considered: normal for values up to 3 mm; overjet for values greater than 3 mm; and anterior crossbite when the incisors were in an inverted positioned with the lower incisal edge occluding buccally to the upper incisal edge.

Overbite: Obtained by measuring the vertical distance between the upper and lower central incisor edges with the teeth in occlusion. This distance was considered: normal when the upper incisors covered the lowers up to 3 mm; and overbite for values greater than 3 mm. When there was no overlap between the upper and lower incisors, with a minimum space of 1 mm between both incisal edges, it was considered open bite.

Posterior crossbite: It was considered present when, in occlusion, the buccal cusps of the mandibular molars were buccally displaced regarding the buccal cusps of the upper molars. Only its presence or absence was considered, regardless of the side.

### Statistical analysis

Data were analytically processed by descriptive statistics and association analysis between the variables collected by the maternal report and exam results. The chi-square (*χ*^2^) or Fisher’s exact test with the aid of the “BioEstat 5.0” program [20] was performed, with a level of significance of 5% (p < 0.05). In particular, the Fisher’s exact test was used when the statistical program considered the chi-square inappropriate. In such cases, the value of *χ*2 was discarded, keeping only the "p" value from the Fisher’s exact test.

## Results

Table 1 shows the habits prevalence during the monitoring period. Bottle feeding was the most prevalent habit at 12, 18 and 30 months (87.5%; 90% and 96.25%, respectively), followed by pacifier with 42.5% at 12 months and 38.75% at 18 and 30 months. Breastfeeding, who at 12 months had a low percentage (40%), dropped to only 12.50% at the end of the survey.

**Table 1:** Habits at 12, 18 and 30 months after birth in children living in the southeast of Brazil

**Habits**	**12 months**		**18 months**		**30 months**	
	**n**	**%**	**n**	**%**	**n**	**%**
Pacifier sucking	34	42.50	31	38.75	31	38.75
Finger sucking	12	15.00	10	12.50	9	11.25
Nocturnal mouth breathing	11	14.75	12	15.00	17	21.25
Bottle feeding	70	87.50	72	90.00	77	96.25
Breastfeeding	32	40.00	20	25.00	10	12.50

Nearly 70% of the children in this study had some sort of malocclusion. The diagnosis at 30 months is shown in Table 2. Overjet prevailed over the normal condition. Together, open bite and overbite also prevailed over the normal condition. Posterior crossbite was present in fewer than half of the sample.

**Table 2:** Malocclusions at 30 months after birth in children living in the southeast of Brazil

**Overjet**	**n**	**%**
Normal (up to 3 mm)	34	42.50
Overjet	46	57.50
Anterior crossbite	0	0.00
**Overbite**	**n**	**%**
Open bite	27	33.80
Normal (up to 3 mm)	38	47.50
Overbite	15	18.80
**Posterior crossbite**	**n**	**%**
Presence	34	42.50
Absence	46	57.50

When associating habits and malocclusion (Table 3), it was observed that children with finger or pacifier sucking habits had a higher prevalence of overjet (p = 0.028, p = 0.010, p = 0.017, for finger at 12, 18 and 30 months, respectively; and p < 0.0001 for pacifier at 3 periods) and open bite (p = 0.0008, p = 0.003, p = 0.0008, for finger at 12, 18 and 30 months, respectively; and p = 0.0003 at 12 and 18 months, and p < 0.0001 at 30 months for pacifier). The pacifier sucking habit at 30 months was also associated with overbite (p = 0.002). The posterior crossbite was associated with children who were bottle fed at 12 and 30 months (p = 0.02 and p = 0.04, respectively), and who were nocturnal mouth breathers at 12 and 18 months (p = 0.012 and p = 0.005, respectively). Breastfeeding had a significant statistical association with overjet (p < 0.0001 at 12 and 18 months, and p = 0.01 at 30 months) and open bite (p = 0.0002, p = 0.001 and p = 0.01 at 12, 18 and 30 months, respectively).

**Table 3:** Association between habits at 12, 18 and 30 months and malocclusions at 30 months after birth in children living in the southeast of Brazil

**Habit**	**Months after birth**	**Overjet**		**Overbite**		**Open bite**		**Posterior crossbite**	
		***χ*2**	**p value**	***χ*2**	**p value**	***χ*2**	**p value**	***χ*2**	**p value**
Pacifier sucking	12	22.8	*<0.0001	nc	0.058	12.9	*0.0003	0.7	0.538
	18	22.8	*<0.0001	nc	0.058	12.9	*0.0003	0.7	0.538
	30	26.9	*<0.0001	9.3	*0.002	17.1	*<0.0001	1.7	0.18
Finger sucking	12	nc	*0.028	nc	0.865	nc	*0.0008	1.4	0.4
	18	nc	*0.010	nc	0.745	nc	*0.003	nc	0.12
	30	nc	*0.017	nc	0.865	nc	*0.0008	nc	0.23
Nocturnal mouth breathing	12	nc	0.153	nc	0.715	2.4	0.219	nc	*0.012
	18	0.4	0.703	nc	0.716	1.6	0.337	nc	*0.005
	30	1.5	0.34	nc	0.6	1.7	0.308	4.3	0.07
Bottle feeding	12	nc	0.06	nc	0.4	0.9	0.32	4.9	*0.02
	18	nc	0.46	nc	0.63	0.3	0.58	1.1	0.29
	30	nc	0.61	nc	0.35	nc	0.73	4.2	*0.04
Breastfeeding	12	23.05	*<0.0001	3.07	0.07	14.17	*0.0002	2.7	0.09
	18	19.7	*<0.0001	nc	0.2	9.85	*0.001	1.7	0.19
	30	nc	*0.01	nc	0.91	nc	* 0.01	0.7	0.39

(* = statistical significance/nc = discarded *χ*2).

Table 4 shows the frequency of overjet and open bite at 30 months, and breastfeeding at 12, 18 and 30 months, to help in understanding the association between breastfeeding and these malocclusions.

**Table 4:** Overjet and open bite at 30 months, and breastfeeding at 12, 18 and 30 months after birth in children living in the southeast of Brazil

**Breastfeeding**		**Malocclusion**							
		Overjet*				**Open bite**			
		**yes**		**no**		**yes**		**no**	
		**n**	**%**	**n**	**%**	**n**	**%**	**n**	**%**
12 months	yes	8	17.39	24	70.59	3	11.11	29	54.72
	no	38	82.61	10	29.41	24	88.89	24	45.28
	total	46	100.00	34	100.00	27	100.00	53	100.00
18 months	yes	3	6.52	17	50.00	1	3.70	19	35.85
	no	43	93.48	17	50.00	26	96.30	34	64.15
	total	46	100.00	34	100.00	27	100.00	53	100.00
30 months	yes	2	4.35	8	23.53	0	0.00	10	18.87
	no	44	95.65	26	76.47	27	100.00	43	81.13
	total	46	100.00	34	100.00	27	100.00	53	100.00

(* > 3 mm).

## Discussion

Sucking habits have been reported as one of the factors for the onset of malocclusion [21]. Half of the mothers interviewed in a study said that “children crying at night” was the most important reason for acquiring non-nutritive sucking habits [21]. Some situations may stimulate sucking habits, including hunger, fear, physical and emotional stress [22]. During the period of this study, which followed 80 children from the 12^th^ to the 30^th^ month after birth, it was observed that all of them had at least one sucking habit. Moreover, it was observed that, once acquired, habits remained for the majority of children (Table 1). These observations were made possible by the character of this longitudinal study. Almost all studies showed a habit prevalence of less than 100% [6,21,23]. This may happen due to the transversal methodology of most of them, which records a single condition in time and space.

The most prevalent in the three periods evaluated (12, 18 and 30 months) was the bottle (87.5%; 90% and 96.25%, respectively), followed by the pacifier with 42.5% at 12 months and 38.75% at 18 and 30 months. A survey by the Brazilian Ministry of Health in 2009 [23] showed a 58.4% prevalence of bottle use in the Brazilian territory for children less than 12 months of age. The southeast region presenting the largest percentage (63.8%) [23]. Regarding pacifiers, the same survey showed a national rate of 42.6%; 50.3% in the Southeast - the second highest region. The literature show a lot of controversy on the use of pacifiers: if, on one hand, it is associated with the genesis of oral problems, on the other hand, it has shown a potential protection against Sudden Infant Death Syndrome (SIDS) [24].

Nearly 70% of the children in this study had some kind of malocclusion. This rate is a little above the 60% described by Tomita et al. [25] and Gimenez et al. [26] and is similar to the findings by Silva-Filho et al. [18] and Dimberg et al. [19], which were 73% and 70%, respectively.

As for the prevalence of malocclusion, the main condition found was the overjet, present in more than half the sample (57.5%), followed by posterior crossbite (42.5%) and open bite (33.75%). These figures differ slightly from those reported by Gimenez et al. [26], in which the main condition found was open bite, followed by overjet and anterior crossbite. On the other hand, Heimer et al. [3] reported 32.1% for open bite and 26% for posterior crossbite. Peres et al. [5] found the prevalence of 46.2% for open bite and 18.2% for posterior crossbite in southern Brazil.

Pacifier sucking at 12, 18 and 30 months of age was associated with overjet and open bite; and at 30 months, an association with overbite was also observed. These results corroborate other studies, like those of Katz et al. [9] and Gimenez et al. [26]. Children with finger sucking habit at 12, 18 and 30 months also had more overjet and open bite. One study showed that if pacifiers and finger sucking habits exceed 48 months of age, the induction potential of malocclusion increases considerably [10]. In addition, finger sucking has a potential similar to pacifier use to induce malocclusion when these habits persist beyond that age [10]. However, malocclusions could have a physiological resolution if the deleterious habit is interrupted until 4 years of age, because until this age the body has the capacity for self-correction of malocclusion [27]. This spontaneous correction may have different rates according to the practiced sucking habit [19].

The posterior crossbite was more prevalent for children who slept breathing through their mouth at ages 12 and 18 months. Several studies have associated mouth breathing with malocclusion [11,12,28]. Accordingly, Mocellin et al. [29] found that 63.3% of people with atretic palate and posterior crossbite were mouth breathers. The bottle feeding habit also was associated with posterior crossbite. This association occurred at 12 and 30 months. Montaldo et al. [30] reported that children with bottle feeding showed a greater risk of crossbite.

According to the WHO [31], breastfeeding should be exclusive until the sixth month of age and supplemented until two years old or later. Data and results about sucking habits for this population under the age of 12 months were reported in a previous article that found that only 50% of children were exclusively breastfed in the first month, and none were exclusively breastfed at 6 months of age [32]. In this research, breastfeeding had significantly statistical association with overjet and open bite at 12, 18 and 30 months. When observing Table 4, it can be seen that the great majority of children who presented overjet and open bite at 30 months, had been totally weaned at 12 months (82.61% and 88.89%, respectively). These percentages increased during the research, reaching 95.65% and 100.00%, respectively, at 30 months. This suggests that the association found may have been due to low breastfeeding rates, which may have contributed to the development of harmful habits. Some studies indicate that breastfeeding can prevent the occurrence of sucking habits [33-35], and others have demonstrated the preventive benefits of breastfeeding on the misalignment of the arcades [26,36]. The lack or absence of breastfeeding is correlated to the underdevelopment of the masticatory complex, the onset of mixed or mouth breathing, tongue thrusting, search for other habits and, therefore, inappropriate development leading to malocclusion [26].

Bottle feeding influences the oral sensory-motor system by producing less muscular involvement [37]. It causes decreased jaw work, causing vacuum sucking movements with the tongue, lips and cheeks, which may lead the tongue to press the nipple against the palate, thus generating a high palate and crossing the bite in the posterior region [37]. This was observed in this study. Children who used the bottle at 12 and 30 months showed a higher prevalence of posterior crossbite.

The number of children with harmful habits, even with the mothers having received guidance on their risk for oral health, proved to be a problem that needs more discussion. In general, the habits were associated with signs of malocclusion, as reported in the literature. However, studies with larger samples and with infants exclusively breastfed for longer periods can perhaps detect more associations. The mother’s reports may be considered a possible bias, and to avoid this bias, follow-up home visits were made. Moreover, it would be important to conduct further studies with this population, extending to the mixed dentition to verify if the observed associations persist and seek other possibilities.

## Conclusions

Sucking habits and nocturnal mouth breathing were predisposing to malocclusion. Children with a finger sucking habit, as well as those with low rates of breastfeeding, were more susceptible to overjet and open bite. Children with a pacifier sucking habit were more susceptible to overjet, open bite and overbite. Posterior crossbite was associated with bottle fed children and nocturnal mouth breathers.

## Abbreviations

UNESP: São Paulo State University; WHO: World Health Organization; SIDS: Sudden infant death syndrome.

## Competing interests

The authors declare that they have no competing interests.

## Authors’ contributions

SASM had the original idea, designed the study and participated in the writing. AJIG and CASG participated in the writing. AMCL participated in the final writing. LFL designed the study, collected data and wrote the first version of the article. OS conducted the statistical analysis. All authors interpreted the data; collaborated in the article's revision and critical analysis; and approved the final manuscript.

## Pre-publication history

The pre-publication history for this paper can be accessed here:

http://www.biomedcentral.com/1472-6831/14/96/prepub
